# Meteorological Table

**Published:** 1813-06

**Authors:** 


### METEOROLOGICAL TABLE.

*From April 25, to May 25, 1813.*

| | D. | Therm. | | | Barom. | | Hygrom. Dry.— | | | —Damp. | | | Winds. | Atmos. Variation. |
|---|---|---|---|---|---|---|---|---|---|---|---|---|---|---|
| | 26 | 46 | 57 | 47 | $29^{8}$ | — | 20 | — | 23 | | | | SW. | C. F... |
| | 27 | 47 | 48 | 49 | $29^{6}$ | 4 | 16 | 12 | 10 | | | | W. | R... |
| | 28 | 45 | 50 | 46 | $29^{4}$ | 6 | 10 | — | — | | | | SE. | C... R. |
| | 29 | 44 | 45 | 43 | $29^{7}$ | — | 14 | — | 12 | | | | E. | C.. |
| ● | 30 | 42 | 48 | 47 | $29^{6}$ | 7 | 9 | 5 | 2 | | | | E. | R.. C.. |
| | 1 | 48 | 51 | 49 | | | 5 | — | 3 | | | | SE. | R.. —. C.. |
| | 2 | 49 | 54 | 50 | $29^{8}$ | — | | | | | | | E.N | C.. F... |
| | 3 | 55 | 68 | 56 | | | 6 | 10 | — | | | | E... | F.. R.. |
| | 4 | 56 | 66 | 59 | $29^{9}$ | — | 2 | — | — | — | 3 | 4 | NE | F.. R... F.. |
| | 5 | 58 | 66 | 59 | 30 | — | 1 | — | — | | | | N. | F... |
| | 6 | 59 | 67 | 56 | $29^{9}$ | 8 | 4 | 6 | — | — | — | 5 | E... | F... R.... |
| ◐ | 7 | 58 | 69 | 54 | — | — | | | | 4 | — | 1 | NE. | F.. R.. F.... |
| | 8 | 55 | 68 | 56 | $29^{7}$ | — | 1 | — | — | | | | W | F... |
| | 9 | 56 | 71 | 59 | | | | | | 1 | 2 | — | W | F.... |
| | 10 | 59 | 69 | 58 | $29^{9}$ | — | | | | 5 | 6 | — | W | F... R.. F... |
| | 11 | 59 | 66 | 59 | $29^{8}$ | — | | | | 5 | 4 | 3 | W | F... R.. F... |
| | 12 | 58 | 69 | 56 | $29^{7}$ | — | 2 | 10 | 1 | | | | NW | F.... |
| | 13 | 57 | 66 | 56 | $29^{6}$ | — | 5 | 15 | 6 | | | | NW | F... |
| | 14 | 57 | 64 | 55 | $29^{5}$ | — | 4 | 10 | 3 | | | | W | F... |
| ○ | 15 | 56 | 60 | 53 | $29^{6}$ | 7 | 1 | 7 | 3 | | | | NW | C.. R..... F.. |
| | 16 | 53 | 60 | 52 | $29^{5}$ | — | 1 | — | 2 | | | | W | C.. R... R. |
| | 17 | 54 | 58 | 53 | $29^{7}$ | 9 | 4 | 7 | 12 | | | | SW | C.. R.. C... |
| | 18 | 53 | 57 | 54 | $29^{9}$ | — | 7 | 9 | 3 | | | | W | C.. R. C.. |
| | 19 | 54 | 59 | 55 | $29^{8}$ | 7 | 1 | — | — | | | | W | C.. R.. F. |
| | 20 | 53 | 57 | 49 | $29^{6}$ | 5 | 4 | 2 | 1 | | | | NW | C.. R... C.. |
| | 21 | 48 | 53 | 44 | — | — | 1 | — | — | | | | NW | F...R....Hail...C.. |
| | 22 | 47 | 54 | 50 | $29^{6}$ | 7 | 3 | 9 | 4 | | | | W | C.. R.. C.. |
| ◑ | 23 | 52 | 54 | 54 | $29^{6}$ | 5 | 3 | — | — | — | 1 | 2 | W | C.. R. R. |
| | 24 | 54 | 58 | 51 | $29^{6}$ | — | 4 | 7 | 9 | | | | W | C. R.. F.... |
| | 25 | 55 | 56 | 54 | | | 5 | 9 | 2 | | | | W | C. R.. C.. |

Quantity of rain from the 25th of April, to the 25th of May, 3 inches $\frac{20}{100}$.

May has been by far the wettest month of the present year; many showers of hail have also fallen. On the evenings of the 3d and 4th, serene lightning; and in the early part of the night of the 6th, a severe storm of thunder, falling with great violence on the metropolis and adjacent villages; and in the latter some mischief was done by the lightning.

Notwithstanding the dampness and coldness of the present season, diseases are assuming their summer form. Some cases of what is commonly called Typhus have appeared. Quick and not strong pulse, no very great increase of heat, with black sordes collecting on the teeth and tongue, with slight delirium, and considerable prostration of strength, have characterised this fever; but we have not learned with any certainty that it was received or disseminated by contagion. In some instances it has been fatal; and we have to lament that a young surgeon of great promise, *Mr. Ogborne,* of Great Portland Street, has been one of its victims.

*Prince's Street, Cavendish Square.*

